# Block Copolymer Template-Directed Catalytic Systems: Recent Progress and Perspectives

**DOI:** 10.3390/membranes11050318

**Published:** 2021-04-27

**Authors:** Labeesh Kumar, Sajan Singh, Andriy Horechyy, Andreas Fery, Bhanu Nandan

**Affiliations:** 1 Leibniz-Institut für Polymerforschung Dresden e. V., Hohe Str. 6, 01069 Dresden, Germany; kumar-labeesh@ipfdd.de; 2 Department of Textile Technology, Indian Institute of Technology Delhi, Hauz Khas, New Delhi 110016, India; sajansingh1507@gmail.com; 3 Institute of Physical Chemistry of Polymeric Materials, Technische Universität Dresden, 01062 Dresden, Germany

**Keywords:** block copolymer, self-assembly, nano-catalyst, micelles, core-shell, yolk-shell

## Abstract

Fabrication of block copolymer (BCP) template-assisted nano-catalysts has been a subject of immense interest in the field of catalysis and polymer chemistry for more than two decades now. Different methods, such as colloidal route, on-substrate methods, bulk self-assembly approaches, combined approaches, and many others have been used to prepare such nano-catalysts. The present review focuses on the advances made in this direction using diblock, triblock, and other types of BCP self-assembled structures. It will be shown how interestingly, researchers have exploited the features of tunable periodicity, domain orientation, and degree of lateral orders of self-assembled BCPs by using fundamental approaches, as well as using different combinations of simple methods to fabricate efficient catalysts. These approaches allow for fabricating catalysts that are used for the growth of single- and multi-walled carbon nanotubes (CNTs) on the substrate, size-dependent electrooxidation of the carbon mono oxide, cracking of 1,3,5-triisopropylbenzene (TIPB), methanol oxidation, formic acid oxidation, and for catalytic degradation of dyes and water pollutants, etc. The focus will also be on how efficient and ease-of-use catalysts can be fabricated using different BCP templates, and how these have contributed to the fabrication of different nano-catalysts, such as nanoparticle array catalysts, strawberry and Janus-like nanoparticles catalysts, mesoporous nanoparticles and film catalysts, gyroid-based bicontinuous catalysts, and hollow fiber membrane catalysts.

## 1. Introduction and Scope

In recent years, a lot of interest has been shown by researchers in the field of block copolymer (BCP)-assisted synthesis of functional (nano)materials for various applications, such as membranes [1,2], catalysis [3,4,5], drug delivery [6,7,8,9], gas absorption [10], etc. The microphase-separated BCPs are known to form periodic nanostructures with different degrees of complexity, ranging from the simplest diBCP morphologies (spherical, cylindrical, gyroidal, or lamellar structures), up to the very complex and sometimes unique nanostructures like “knitting patterns”, multi-helix, or helical torroids [11,12,13]. An extremely large spectrum of self-assembled BCP morphologies with tuneable periodicity, domain orientation, and degrees of lateral order provide enormous potential for the synthesis of various functional (nano-)materials with targeted characteristics and properties. To date, the vast variety of organic (polymeric), hybrid organic/inorganic, and purely inorganic nanostructures have been created to explore self-assembled BCPs as templates. These nanostructures, which often replicate the initial morphology of the BCP matrix, have been further used in different application areas.

**Figure 1:**
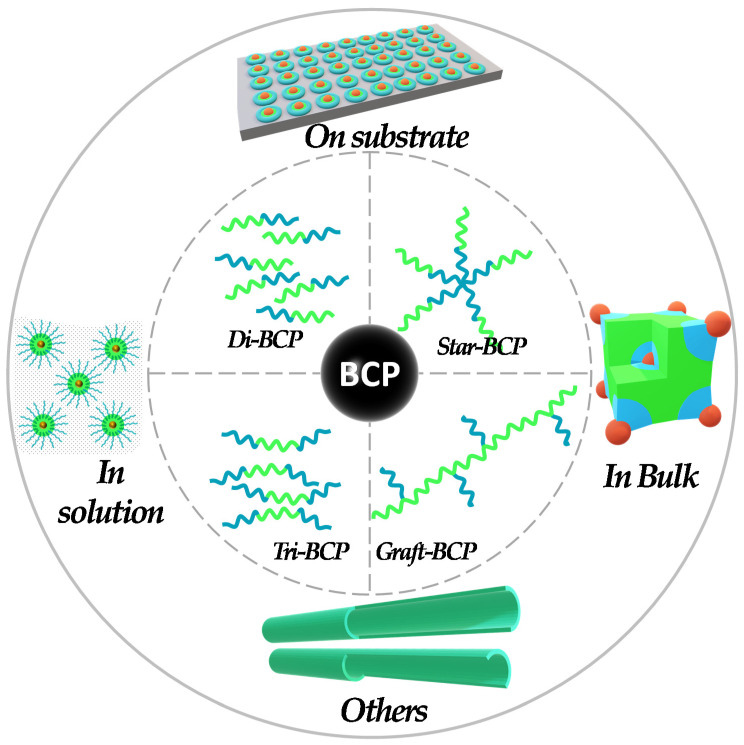
BCP template-based approaches used for the fabrication of (nano-)catalysts.

BCP self-assembly has been proven as a powerful and versatile tool to direct periodic morphology, porosity, structural features, and functionality for organic and inorganic nanostructures. For this reason, BCPs have gained the highest popularity in the field of templates, in order to provide numerous nanomaterial architectural templates using distinct methods. The classification of the pathways exploiting the BCP templates in the preparation of catalysts can be broadly classified into approaches like on-substrate templating, colloidal templating, and bulk templating (Figure 1). However, as will be shown in the next section, most of the work reported in this study basically utilizes a combination of these approaches. The first three are the basic approaches, which researchers have utilized in few cases, but interestingly, the combination of these basic approaches have been more popular among researchers. 

In this review article, our focus is on the block copolymer template-directed nanostructured catalytic systems that have been reported in literature in recent years. The whole-spectrum BCP compositions that have been used for the fabrication of catalysts are very bright, whereas the BCPs discussed in present review paper consist of the following segments: poly(acrylic acid) (PAA), poly(2-vinyl pyridine) (P2VP), poly(4-vinyl pyridine) (P4VP), poly(ethylene glycole) (PEG), poly(ethylene oxide) (PEO), poly(propylene oxide) (PPO), poly(L-lactide) (PLLA), and poly(ferrocenyldimethylsilane) (PFS). 

## 2. BCP Templates for Catalysts

BCPs have been widely used as templates for patterning of various inorganic materials on the nanoscale. The past work done for templating catalytic materials using block copolymer self-assembly can be broadly categorized in two sections, i.e., micellar/colloidal assembly or the on-substrate approach, as well as bulk approaches as detailed below in the given sub-sections. 

### 2.1. Micellar/Colloidal Assembly and/or on-Substrate Approach

BCP micellar template approach has been one of the most frequently utilized approach for templating catalytic materials. BCP micelles were used to accommodate various precursors and/or presynthesized nano-catalysts in specific domains, and also provides a low-cost option to deposit thin film on substrates, such as via evaporation and sputtering. BCPs having blocks capable of coordinating with metals or metal ions, like P4VP or P2VP [14,15,16,17], have been most frequently used for the templating of inorganic materials. The slow exchange kinetics in BCPr micelles allows an easy transfer of the micelles from colloidal solution onto a substrate by exploring different deposition methods, such as spin-coating or dip-coating. Hence, utilizing BCP micellar templates, periodic nanoparticle arrays can be generated on various substrates, whereas additional post-treatment strategies, like thermal or solvent vapor annealing or surface reconstruction, can be implemented to generate nanoparticle arrays with a long-range lateral order. Using the BCP micellar template approach, one can fabricate ordered arrays of nanoparticles (NPs) of desired size and control the interparticle spacing by selecting the BCP of suitable molecular weight, length of the block segments, or using different solvents [18]. Therefore, the choice of BCP determines the areal density of NPs on the substrate. Researchers also utilizes this strategy to fabricate binary and ternary nanoparticle arrays, core-shell and alloy nanoparticle arrays, and torroids and nanowires on the substrate, which could be used for various catalytic applications.

**Figure 2:**
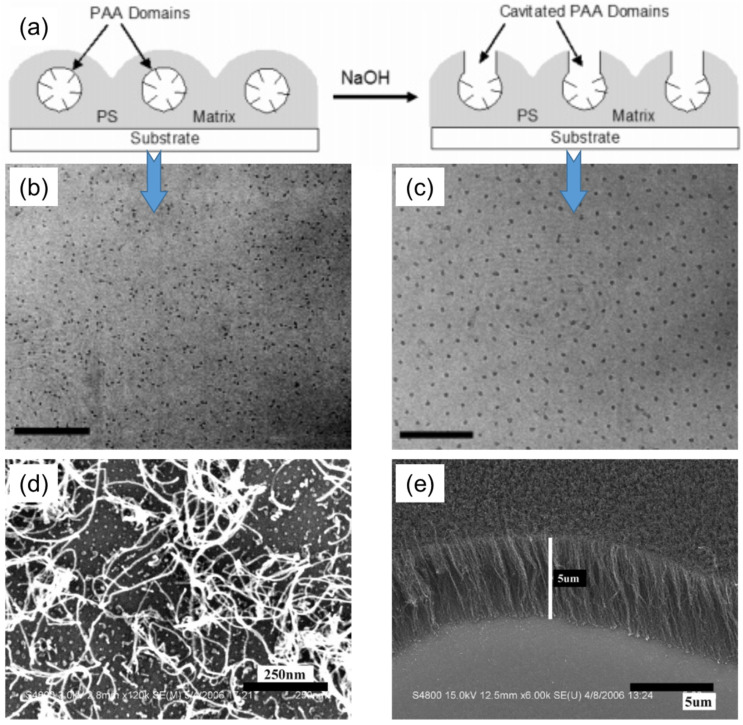
(**a**) Cavitation of PAA domain by submerging PS–*b*–PAA micellar arrays into NaOH aqueous solution. (**b**,**c**) TEM images of iron oxide nanoclusters synthesized using (**b**) non-cavitated template (scale bar = 200 nm), and (**c**) cavitated template (scale bar = 150 nm). Adapted from [21]. (**d**,**e**) SEM images of CNTs grown on Si substrate decorated with (**d**) low-density (1400/µm^2^), and (**e**) high-density (3800/µm^2^) particle arrays. Adapted from [22].

Jamamillo et al. reported the first example of catalytic application of gold (Au) NPs synthesized by the BCP micelle template approach for electro-oxidation of carbon monoxide [19]. Here, the Au nanoclusters of tightly controlled particle size were synthesized by a simple dip-coating of the substrates, with Au precursors encapsulated by BCP micelles, followed by the conversion of AU precursors to form Au NP arrays on the substrate. Later on, the BCP micellar templating strategy was extensively used for the fabrication of various other nano-catalysts. Here, we provide several examples of nano-catalysts derived from BCP micellar templates. Benett et al. reported catalytic growth of CNTs from the arrays of iron oxide nanoclusters prepared using BCP micellar templates [20]. Boontongkong et al. showed that PAA cores of PS–*b*–PAA BCP micelles are well-suited for the lodging of aprotic ionic metal salts, which are useful for carbon nanotube growth [21]. The toluene solubilized PS–*b*–PAA micelles were first deposited onto the substrates by spin-casting, and then submerged in NaOH solution, which induced the swelling of the PAA micellar cores. The core swelling caused the rupture of PAA domains to the surface and formation of cavities, as schematically shown in Figure 2a. The cavitated thin film was further submerged in FeCl_3_ solution to directly exchange Na^+^ to Fe^3+^ ions. The polymeric part was then degraded upon heat treatment, which resulted in iron oxide nanocluster arrays arranged into an ordered hexagonal pattern on the substrate. The cavitation step was decisive in order to obtain hexagonally ordered iron oxide clusters (Figure 2b,c). These arrays were further used as catalytic substrate for the synthesis of CNTs, using thermal CVD in a quartz-tube reactor. Liu et al. further explored the approach given by Bennett et al., and showed that vertically aligned CNTs can be grown from the substrate by using a high areal density of the catalyst particles (Figure 2d,e) [22].

**Figure 3:**
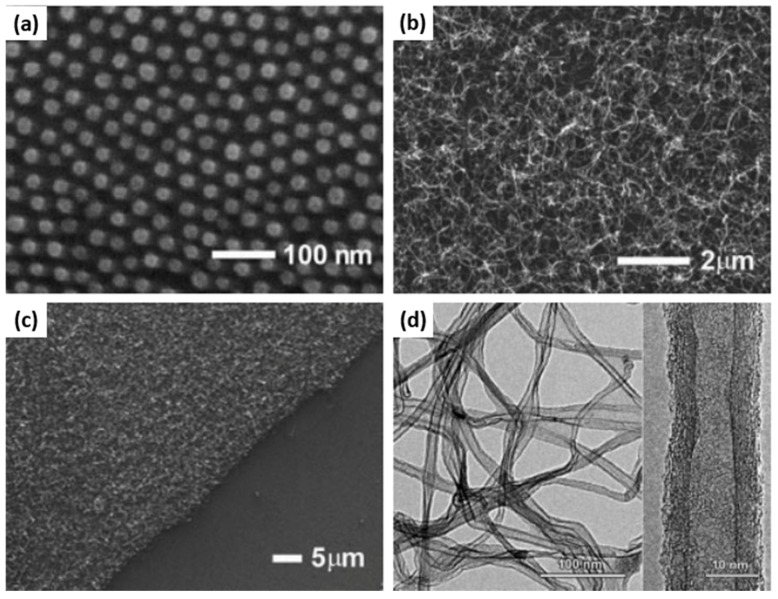
(**a**) SEM image iron oxide domains on a Si surface obtained by spin-coating a PS–*b*-PFS block copolymer followed by O_2_ reactive-ion etching; (**b**) SEM image of the same surface after CNT growth for 5 min. (**c**) SEM image of the same surface as (**b**). In the lower righthand corner the polymer film was scratched before O_2_ reactive-ion etching. (**d**) Medium- and high-resolution TEM images of carbon nanotubes grown as in (**b**,**c**). Adapted from Ref. [23].

Hinderling et al. reported another BCP templating method to grow CNTs from the catalyst-decorated substrate employing the iron-containing PS–*b*–PFS BCP [23]. The size and composition of BCP was chosen in such a way that it formed hexagonally ordered PFS spherical domains in the PS matrix. The PS–*b*–PFS thin films deposited on silicon substrate were thermally annealed in vacuum and then treated in an oxygen plasma to remove PS matrix and convert PFS domains into well-ordered NPs arrays (Figure 3a). The CNTs were grown in a thermal CVD reactor on this substrate, using acetylene as a carbon source, which resulted in a dense coverage of the substrate with multiwalled CNTs (Figure 3b). The CNT growth was not observed in the regions from where the polymer film was removed before O_2_ plasma treatment (Figure 3c). This demonstrated the catalytic activity of fabricated nanoparticle array. TEM imaging of CNTs revealed the crystallinity of the graphitic walls and some amorphous carbon on the nanotubes (Figure 3d). 

**Figure 4:**
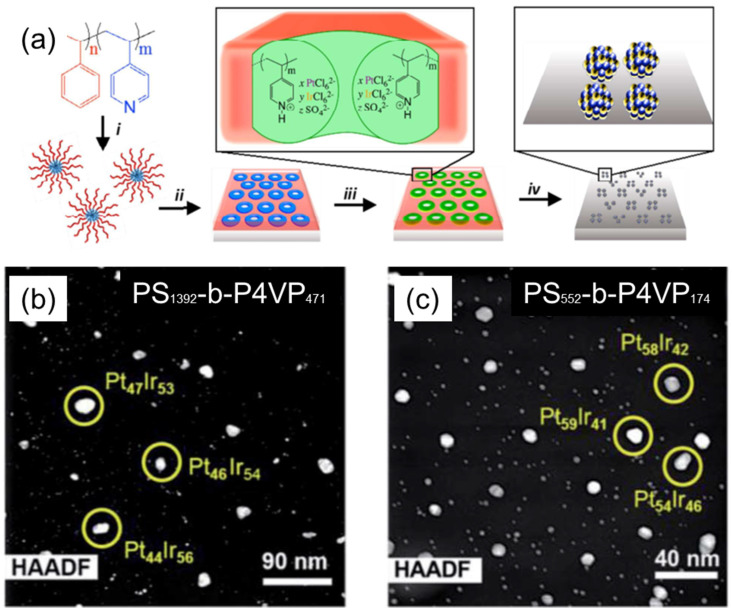
(**a**) Schematics of the synthetic procedure for the synthesis of PtIr bimetallic catalysts, using thin films of PS–*b*-P4VP micelles: (i) solution self-assembly of PS–*b*–P4VP into spherical micelles in toluene; (ii) spin-coating micelles onto an electrode substrate, followed by thin-film reconstruction into an array of inverted micelles by soaking in methanol; (iii) simultaneous loading of H2PtCl6 and H_2_IrCl_6_ precursors into the P4VP domains; and (iv) thermoreductive annealing under Ar flow to remove PS–*b*–P4VP template and convert metal precursors into PtIr alloy NPs; (**b**,**c**) HAADF STEM images of PtIr NPs arrays derived from (**b**) PS_1392_-*b*–P4VP_471_ and (**c**) PS_552_-*b*–P4VP_174_ templates loaded with H_2_PtCl_6_ + H_2_IrCl_6_ at a stoichiometric ratio of 8:2 mol/mol. Subscripts in BCP assignments correspond to the number of monomer repeat units of each block. Adapted from [24].

Taylor et al. have demonstrated the synthesis of PtIr bimetallic alloy catalysts using thin films of (PS–*b*–P4VP) inverted micelles, as shown in Figure 4a [24]. PS–*b*–P4VP micelles were first deposited onto the substrate and then exposed to methanol to create PS–*b*–P4VP inverted micelles. Pt and Ir ions were simultaneously loaded into P4VP domains by immersing the substrate into an acidified aqueous solution containing H_2_PtCl_6_ and H_2_IrCl_6_ metal precursors. The thermoreductive annealing performed at 600 °C under Ar flow simultaneously degraded the BCP template, reduced the metal anions, and alloyed the resulting metals to form bimetallic PtIr NP arrays. The composition of the PtIr alloy NP could be tuned by the ratio of metal precursors in the loading bath. Interestingly, the relationship between the metal precursor ratio in the loading baths and the Pt/Ir atomic ratio in the synthesized alloy NPs was non-linear. Moreover, the resulting Pt/Ir ratio in synthesized alloy NPs also varied depending on the length of PS–*b*–P4VP BCP used for the preparation of micellar templates (Figure 4b,c). The electrocatalytic activity studies performed by cyclic voltamperometry revealed that PtIr alloy NP arrays are highly active catalysts for formic acid oxidation reactions, with the highest mass activity value of 37 A g^−1^ for the Pt_16_Ir_84_ composition, which is four-fold higher than that of the PtRu industrial research standard.

**Figure 5:**
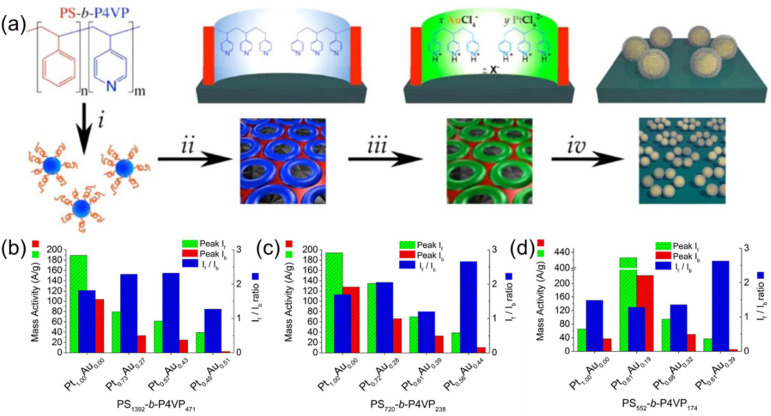
(**a**) Schematics of synthetic procedure for the synthesis of PtAu bimetallic, core-shell catalysts, using thin films of PS–*b*–P4VP micelles. (i) Solution self-assembly of PS–*b*–P4VP into spherical micelles in toluene; (ii) spin-coating micelles onto an electrode substrate, followed by thin film reconstruction into an array of open micelles by soaking in methanol; (iii) simultaneous loading of K_2_PtCl_6_ and KAuCl_4_ metal precursors into micellar array by immersion into a stoichiometrically tuned, aqueous solution of the respective metal ions; (iv) removal of PS–*b*–P4VP template by reductive Ar plasma etching. (**b**–**d**) Electrocatalytic activity charts for peak mass activity, *I_f_* and *I_b_*, and *I_f_*/*I_b_* ratio observed during 50 cycles of methanol oxidation for PtAu core-shell NPs, with varied Pt/Au ratios created from PS_1392_–*b*–P4VP_471_ (**b**), PS_720_–*b*–P4VP_238_, (**c**) and PS_552_–*b*–P4VP_174_ (**d**). Subscripts in BCP assignments indicate the average number of PS and P4VP units in BCP composition, whereas *I_f_* and *I_b_* denote the forward and reverse anodic peak current, respectively, derived from cyclic voltammetry experiments for methanol oxidation. Adapted from [25].

Mikkelsen et al. also used the BCP template approach to fabricate PtAu core-shell bimetallic nano-catalysts with tailored composition, particle density, and electrochemical activity, using thin films of PS–*b*–P4VP micelles, as shown in Figure 5a [25]. The synthesized PtAu bimetallic NPs comprised a Pt-rich shell surrounding a PtAu alloy core. The PtAu core-shell bimetallic NPs were found to have a very high density of electrochemically active Pt surface sites. Accordingly, the activity of Pt-rich, core-shell PtAu nanocatalysts for the electrocatalytic oxidation methanol was approximately 2–4-fold that of a monometallic Pt benchmark catalyst (ETEK), and only 28% less than that of the PtRu bimetallic benchmark catalyst (XC-72R). Moreover, the Au that was present in the PtAu NPs was beneficial to Pt-rich electrocatalysts, as the carbonaceous poisoning *I_f_*/*I_b_* metric was greatly enhanced relative to both benchmark materials (~2–3-fold increase) (Figure 5b–d).

**Figure 6:**
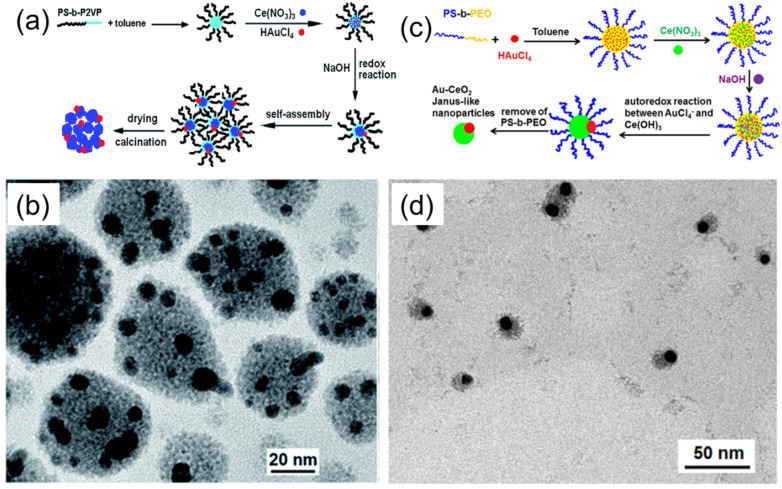
(**a**) Schematic illustration of the process leading to the formation of catalytically active, Au–CeO_2_ strawberry-like particles, using PS–*b*–P2VP/Ce(NO_3_)_3_/HAuCl_4_ composite micelles. (**b**) TEM image of Au–CeO_2_ Janus particles, prepared using PS–*b*–PEO/Ce(NO_3_)_3_/HAuCl_4_ composite micelles underoptimized conditions. Adapted from [26]. (**c**) Schematic illustration of the process leading to the formation of Au–CeO_2_ Janus-like nanoparticles using PS–*b*–PEO/Ce(NO_3_)_3_/HAuCl_4_ composite micelles. (**d**) TEM image of Au–CeO_2_ Janus particles prepared using PS–*b*–PEO/Ce(NO_3_)_3_/HAuCl_4_ composite micelles under optimized conditions. Adapted from [27].

Recently, Jiao et al. have demonstrated the fabrication of strawberry-like Au–CeO_2_ nanoparticles using PS–*b*–P2VP BCP micelles as templates, as schematically shown in Figure 6a [26]. PS–*b*–P2VP/Ce(NO_3_)_3_/HAuCl_4_ composite micelles were prepared by dissolving PS–*b*–P2VP block copolymers, Ce(NO_3_)_3_·6H_2_O, and HAuCl_4_ in a mixture of ethanol and toluene. Both inorganic precursors were incorporated in P2VP cores of the micelles. Upon addition of NaOH to this composite micelle solution, Ce^3+^ ions first reacted with OH^−^ to form Ce(OH)_3_, and sequentially, the auto redox reaction between Au(III) and Ce(III) took place to form Au(0) and Ce(IV). During the process of reaction, the aggregation of hybrid micelles to form larger spheres takes place. As a result, strawberry-like Au-CeO_2_ hybrid particles could be obtained (Figure 6b).

Following a similar idea, Yu et al. have also demonstrated the fabrication of Au–CeO_2_ Janus-like particles [27]. In this approach, hybrid PS–*b*–PEO/Ce(NO_3_)_3_/HAuCl_4_ hybrid micelles were prepared and used as templates for particle formation, as shown in Figure 6c,d. The mechanism of Au–CeO_2_ particle formation was essentially the same as in the case of a PS–*b*–P2VP/Ce(NO_3_)_3_/HAuCl_4_ system. Both inorganic precursors were incorporated in the PEO micellar cores, and the addition of NaOH led to the formation of Ce(OH)_3_ and subsequent auto redox reaction between Au(III) and Ce(IV) species. The morphological difference between the obtained Au–CeO_2_ particles in these two methods is plausibly due to the different colloidal stability of hybrid micelles. Both strawberry-like and Janus Au–CeO_2_ particles were shown to be catalytically active in the degradation of methyl orange under ultrasonic irradiation, and revealed enhanced catalytic activity compared to the reference CeO_2_ or Au–CeO_2_ composite nanoparticles. 

**Figure 7:**
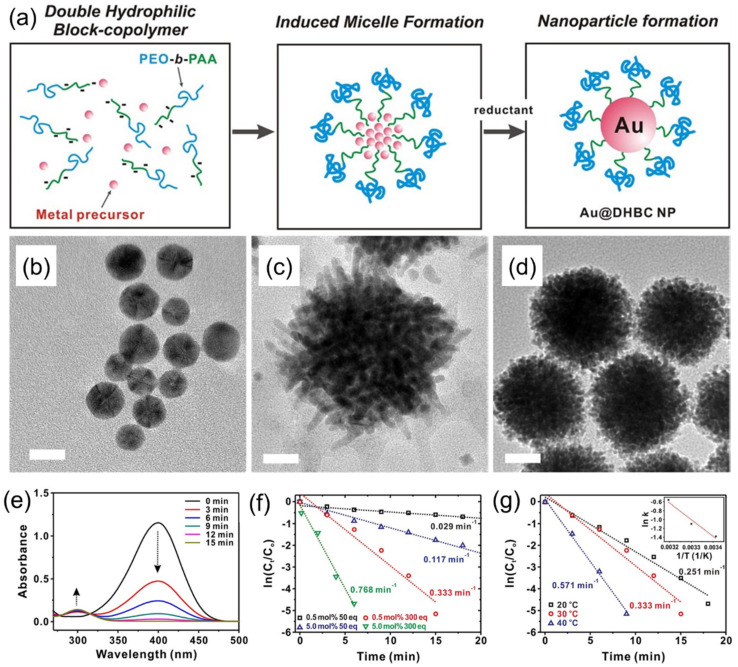
(**a**) Schematics of the synthesis of an Au–DHBC NP through the coordinative bonding between the Au precursor and PEO–*b*–PAA as a soft template. (**b**,**c**) TEM images of (**b**) Au–DHBC, (**c**) Pd–DHBC, and (**d**) Pd–DHBC NPs. Scale bars on TEM images correspond to 10 nm (**b**) and 20 nm (**c**,**d**). (**e**) Time-dependent UV-Vis absorption spectra for the reduction of 4-nitrophenol over an Au–DHBC NP catalyst in aqueous media at 303 K. (**f**) Plot of ln(*C_t_*/*C*_0_) versus time spectra for the reduction of 4-nitrophenol over the Au–DHBC catalyst under different mol% of catalyst and equivalents of NaBH_4_ used, where C_0_ and C_t_ are the initial concentration of 4-nitrophenol and 4-nitrophenol concentration at time *t*, respectively. (**g**) Plot of ln(*C_t_*/*C*_0_) versus time for the reduction of 4-nitrophenol over Au–DHBC NP catalysts under different temperatures at 0.50 mol% catalyst and 300 equiv of NaBH_4_. The inset on (**g**) shows the corresponding Arrhenius plot. Adapted from [28].

Seo et al. also demonstrated a simple approach for synthesizing Au NPs using a double-hydrophilic block copolymer (DHBC) PEO–*b*–PAA as a soft template [28]. Specifically, partially deprotonated, water-soluble PEO–*b*–PAA formed micelles upon the addition of an Au precursor, HAuCl_4_·3H_2_O, which was further converted to AuNPs with the addition of a reducing agent, as schematically shown in Figure 7a. The synthesized DHBC-stabilized Au NPs (Au–DHBC), which appeared in the form of single spherical particles per micelle (Figure 7b), were further used as catalyst for the reduction of 4-nitrophenol. Authors have reported that an AuNP catalyst with the DHBC shell displays a superior catalytic activity in the reduction of 4-nitrophenol compared to Au NPs prepared by citrate reduction, with a similar diameter of AuNPs. Authors demonstrated the versatility of the DHBC-based approach to synthesizing other metal NPs, such as Pd and Pt. Interestingly, the morphology of synthesized Pt–DHBC (Figure 7c) and Pd–DHBC (Figure 7d) was significantly different from the spherical Au–DHBC, though the identical synthetic protocol as described for Au–DHBC was used. The synthesized Au–DHBC was further used to investigate its potential as a catalyst for the reduction of 4-nitrophenol (Figure 7e), where the effect of the catalyst and NaBH_4_ concentrations and the effect of temperature were studied (Figure 7f,g). It was also shown that the Au–DHBC catalyst displays a superior catalytic activity in the reduction of 4-nitrophenol compared to Au NPs prepared by citrate reduction with a similar particle diameter.

**Figure 8:**
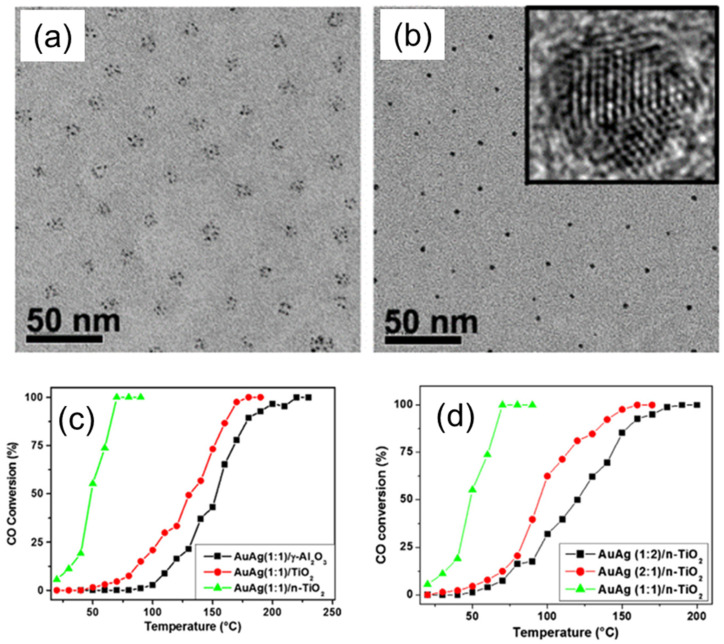
(**a**,**b**) TEM images of the bimetallic AuAg NPs (**a**) before and (**b**) after reduction. The inset shows an HRTEM image obtained for one single NP in (**b**). (**c**,**d**) Catalytic activity of AuAg bimetallic NPs with 1:1 (**c**) and 1:2 (**d**) Au/Ag atomic ratios, deposited on γ-Al_2_O_3_ (black), regular TiO_2_ (red), and BCP templates derived nanostructured n–TiO_2_ during catalytic CO oxidation measured at different temperatures. Reproduced from [29].

In another interesting study, Menezes et al. used PS–*b*–P4VP BCP micelles as nanoreactors for the preparation of stable, bimetallic AuAg NPs, and investigated the effect of particle size, composition, and support on the catalytic activity in the CO oxidation reaction [29]. The synthesis of AuAg NPs was accomplished inside the PS–*b*–P4VP micelles by dissolving BCP in toluene, followed by addition of AgNO_3_ and HAuCl_4_∙H_2_O as metal precursors, and the subsequent reduction of metal precursors with hydrazine monohydrate (N_2_H_4_∙H_2_O). This procedure led to the formation of monodisperse AuAg NPs, with a single NP within each micelle core (Figure 8a,b). For catalytic studies, the AgAu bimetallic NPs were deposited on standard TiO_2_ and nanostructured n–TiO_2_, as well as on γ-Al2O3 supports. The catalytic activity of supported, bimetallic AgAu NPs was significantly higher in case of nTiO_2_ support compared to standard titania and alumina (Figure 8c,d). The authors also investigated the effect of size and composition of AuAg NPs on the catalytic activity in CO oxidation reaction. We demonstrate that at low and moderate temperatures, there is a synergy between the Au and Ag atomic composition of NPs and their catalytic activity, being most pronounced in the case of AuAg compositions with a ratio of 1:1.

**Figure 9:**
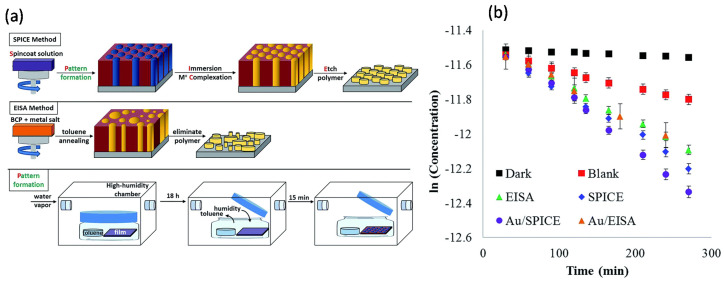
(**a**) The SPICE templating method decouples polymer annealing from the metal precursor gel incorporation. Traditional methods, represented by EISA, spin-coat an all-in-one solution that includes both polymer and metal precursor. Although EISA has fewer steps, it can produce irregular arrays or inorganic material. Pattern formation was achieved by annealing with toluene and water vapor, using standard solvent annealing techniques enclosed in a humidified glove box. (**b**) Plot of the natural log of MB concentration against irradiation time. Error bars represent standard deviations of the mean across three reproducibility studies. Adapted from [30].

Mayeda et al. reported another BCP templating method, i.e., spin-coat pattern–immerse complex etch (SPICE), to produce thin films of well-ordered arrays of metal oxides (MgO, Al_2_O_3_, TiO_2_, Fe_2_O_3_, NiO, etc.) by decoupling metal oxide precursor incorporation from the BCP template, which was otherwise difficult to achieve via the evaporation-induced self-assembly (EISA)/dip-coating approach [30]. Here, perpendicularly oriented and hexagonally packed domains of PEO cylinders in PS–*b*–PEO films were obtained by toluene vapor annealing in a high humidity chamber, as schematically depicted in Figure 9. Subsequently, the templates were immersed in a metal precursor solution, which formed metal–polymer complexes in one polymer domain. Eventually, the organic part was removed in an oxidative environment to leave the templated metal oxides. The SPICE approach was further used to produce ordered TiO_2_ and Au/TiO_2_ arrays, which were subsequently used for the photocatalytic activity to investigate their potential as a catalyst for the degradation of methylene blue (MB). It was found that SPICE–TIO_2_ exhibited a 13% increase in the photocatalytic activity over EISA–TiO_2_. The addition of Au NPs to the SPICE–TiO_2_ samples (Au/TiO_2_) further enhanced the photocatalytic activity by 43%; however, Au NPs did not improve the catalytic activity of the EISA–TiO_2_. 

Prochukhan et al. recently demonstrated a similar but modified, highly scalable approach for fabricating vertical silicon nanotubes arrays using a PS–*b*–PEO, toroidal micelle pattern via a water vapor-induced BCP self-assembly mechanism [31]. The PS–*b*–PEO film readily self-assembled into a toroidal micelle structure on a PS brush surface after spin-coating. Subsequently, the approach was further used for the production of a metal oxide nano-ring array (NiO and Fe_x_O_y_) by integrating metal ion precursors into the PEO corona of the toroidal micelle structure. These structures could be potentially very interesting for catalysis applications.

Ordered mesoporous nanomaterials are the essential nanostructures for catalysis, which can be designed and tuned in different morphologies and different orders and sizes, with adjustable porosities, by using the appropriate compositions and molecular weights of BCP and solvent conditions for block segments. Mesoporous materials comprise a high surface area, and are perfectly suitable to accommodating nano-catalysts in their pores. Hence, they are in high demand in the catalyst field. Recently, several strategies that are based on BCP templating methods have been developed that allow for obtaining mesoporous catalysts.

**Figure 10:**
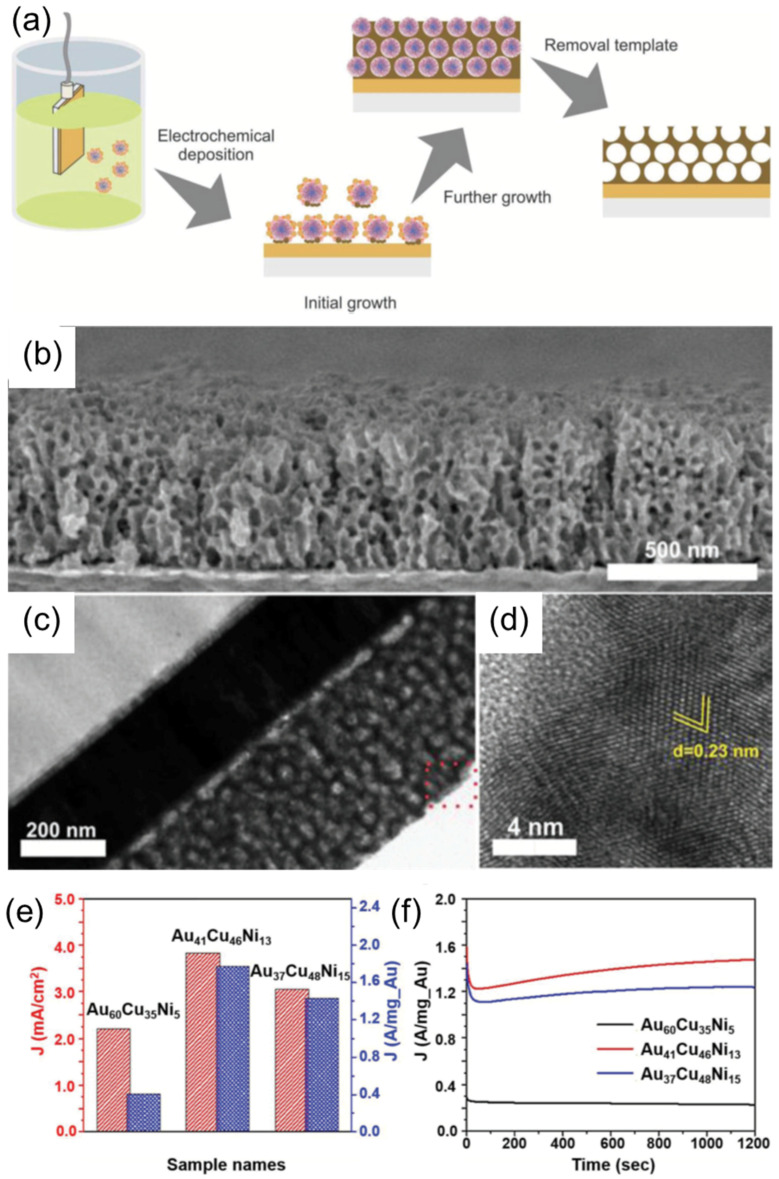
(**a**) Schematic illustration of the electrochemical synthesis of mesoporous, AuCuNi ternary alloy film. (**b**) Cross-sectional SEM image, (**c**) TEM image, and (**d**) high-resolution of TEM image of the mesoporous Au_60_Cu_35_Ni_5_ film obtained at an applied electrodeposition potential of −0.9 V (vs Ag/AgCl) at 40 °C for 1000 s. (**e**) Electrocatalytic activity and (**f**) amperometric response of the mesoporous AuCuNi ternary alloy films with different compositions, recorded at an applied potential of 0.65 V for 1200 s in 1 M NaOH solution + 0.5 M CH_3_OH. Adapted from [3].

Nugraha et al. demonstrated the fabrication of a trimetallic, mesoporous electrocatalyst composed of Au, Cu, and Ni using PS–*b*–PEO BCP micelle multilayers as a template, as shown in Figure 10a–d [3]. The approach involves the co-electrodeposition process of Cu, Ni, and Au precursors pre-loaded into polymeric micelles acting as a sacrificial template. This approach enables accurate control over the dimensional properties and the final film composition by tuning the parameters, such as size of the micelles, electrodeposition time, or metal precursor concentration. Obtained mesoporous structures represent the negative replica of the spherical, multilayer micelle assembles, which remain unaffected upon the removal of P–-*b*–PEO BCP template by solvent extraction using THF. The obtained mesoporous AuCuNi imparted high electrocatalytic activity and stability for methanol oxidation in the alkaline medium (Figure 10e–f).

**Figure 11:**
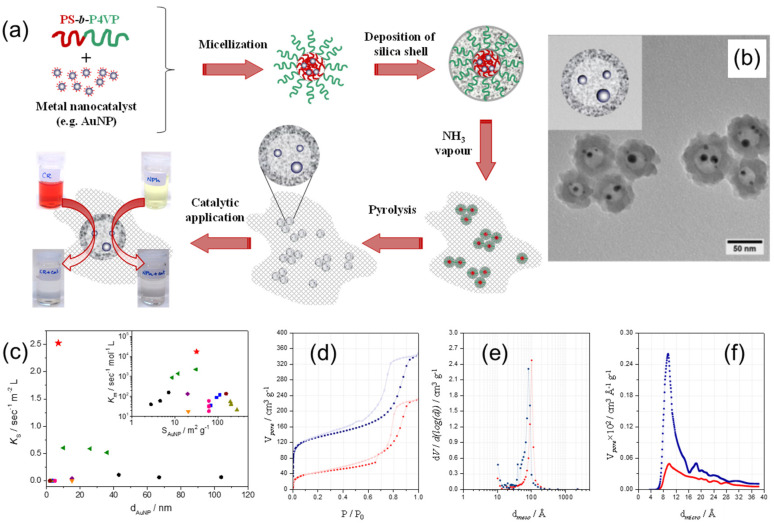
(**a**) Schematics of the preparation of the Au–hollow-SiO_2_–PSS catalyst via the block copolymer template approach; (**b**) TEM image of Au–hollow-SiO_2_ yolk-shell particles, prepared using the BCP template method. (**c**) The comparison of normalized reaction rate constants, *K_s_* and *K_m_* (inset), on the reduction of 4-nitrophenol determined for Au–hollow-SiO_2_–PSS catalyst (red star), with values for analogous catalytic systems reported in literature. (**d**) N_2_ adsorption–desorption isotherms of un-pyrolyzed Au–PS–*b*–P4VP–SiO_2_ (red circles) and pyrolyzed Au–hollow-SiO_2_ particles (blue squares), as well as corresponding size distributions of (**e**) mesopores and (**f**) micropores. Filled and unfilled symbols correspond to adsorption and desorption processes, respectively. Adapted from [32].

Shajkumar et al. reported an interesting approach to prepare an Au–SiO_2_ yolk-shell catalyst embedded in porous silica support (PSS) [32]. Authors used solvent-induced co-assembly process of PS–*b*–P4VP BCP and polymer-stabilized Au nano-catalyst to obtain Au–PS–*b*–P4VP hybrid micelles, followed by a two-step sol gel process to deposit an SiO_2_ shell and generate a PSS matrix, as schematically shown in Figure 11a. The PS–*b*–P4VP BCP directed the structure of the yolk-shell nanoparticles, consisting of Au cores enclosed within the void surrounded by porous silica shell (Figure 11b). The PS core assisted the encapsulation of pre-synthesized AuNP, whereas the protonated P4VP corona helped in the formation of the silica shell. In addition, BCP micelles served as an effective porogen during its removal on pyrolysis. The Au–hollow-SiO_2_–PSS catalyst revealed superior catalytic activity for the catalytic reduction of 4-nitrophenol compared to analogous catalytic systems (Figure 11c) and also good performance in the catalytic degradation of Congo Red dye. The N_2_ adsorption–desorption isotherms of PSS-embedded Au–PS–*b*–P4VP–SiO_2_ core-shell and Au–hollow-SiO_2_ yolk-shell particles, as well as the corresponding size distributions of mesopores and micropores, are shown in Figure 11d–f. After calcination, the surface area increased from 150 to 430 m^2^g^−1^ (or 287%) because of the formation of micropores in the SiO_2_ shell upon removal of the BCP template (Figure 11f). 

### 2.2. Bulk Approaches

**Figure 12:**
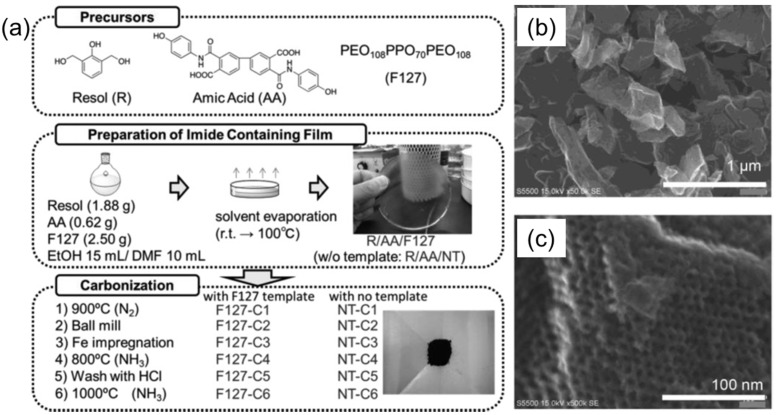
(**a**) Block copolymer-templated carbonization of a nitrogen–containing polymer to create mesoporous carbon for oxygen reduction catalyst. (**b**,**c**) SEM images of F127-C6 mesoporous carbon, taken at different magnifications. Adapted from [33].

Here, the catalytic materials were hosted in the bulk self-assembled structures of BCPs during or after the self-assembly process. Nabae et al. reported the synthesis of ordered, mesoporous carbon materials doped with iron and nitrogen species by employing the crosslinked, self-assembled BCP as the template, and used this material as an electrocatalyst for the reduction of oxygen [33]. The multi-step fabrication procedure is schematically shown in Figure 12a. At first, cross-linked polyimide films were synthesized from a mixture of resol and amic acid, either with or without addition of Pluronic (F127) BCP as template. Subsequently, carbonization, ball milling, Fe impregnation, and NH_3_/HCl etching steps were performed to yield the final carbonous materials, denoted as F127-C6 and NT-6. In contrast to template-free samples, the BCP-templated carbonized samples had an ordered, mesostructured morphology, more than two-fold higher surface area and pore volume, and well-defined and narrowly distributed pores of ca. 4.8 nm. Moreover, the obtained porous carbons were tough enough to retain their mesostructured morphology even after mechanical ball milling (Figure 12b,c). Both F127-C6 and NT-C6 showed catalytic activity for oxygen reduction reaction, and the reaction mechanisms over both catalysts were similar, as indicated by the same onset potential. However, in the case of the F127-C6 catalyst, the diffusion-limiting current was higher as compared to NT-C6, and was attributed to the higher mass transfer of the oxygen molecules in the F127-C6 catalyst.

**Figure 13:**
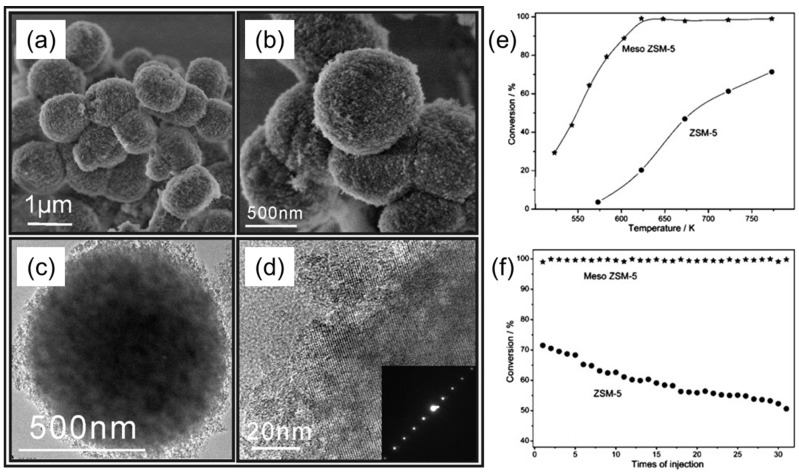
(**a**,**b**) SEM and (**c**,**d**) TEM images of meso ZSM-5 particles. © TEM image of a single meso-ZSM-5 particle. (**d**) HR-TEM image taken at the edge of the particle, showing the continuous lattice stripes with an ED pattern (inset). (**e**,**f**) Catalytic performance of meso-ZSM-5 particles compared to a conventional ZSM-5 catalyst employed in the cracking of TIPB as a probe reaction: (**e**) catalytic activity of meso-ZSM-5 and ZSM-5, determined at different temperatures; (**f**) deactivation behavior at 773 K. Adapted from [34].

Zhou et al. proposed a direct, synthetic strategy to obtain mesoporous zeolites (meso-ZSM-5), using conventional BCP templates with the assistance of post-steaming treatment [34]. They employed the commercially available and low-cost, PEG-based BCP surfactants, such as Pluronics (F127, P123) or a Brij series, as soft templates. At first, the mesoporous template and zeolite precursors were converted into homogeneous gels, which were further dried and crystallized under controlled steaming conditions. In this strategy, BCP templates worked as a scaffold to keep confined space during the conversion of gels to zeolites. The size of mesopores could be tuned simply by changing the amount of meso templates. In Figure 13a,b, SEM images of such uniform, spherical ZSM-5 particles with a rough surface morphology are shown. The TEM and HR-TEM images in Figure 13c,d respectively, represent the development of mesoporosity and the presence of lattice fringe in the framework of meso-ZSM-5, using F127 as the template. This single crystal pattern clearly indicates that the mesoporous network is present inside the spherical particle. A typical probe reaction of the cracking of 1,3,5-triisopropylbenzene (TIPB) was used to analyze the catalytic activity and anti-deactivation properties of meso-ZSM-5 catalysts. The catalytic results in Figure 13e, f reveal that meso ZSM-5 is a better catalyst than the conventional ZSM-5 zeolite within the temperature range tested. In the case of meso-ZSM-5, TIPB conversion increased rapidly with the increase in temperature, and reached 100% at 623 K. In contrary, in the case of conventional ZSM-5, the conversion of TIPB gradually increased from 3.6% at 573 °C to 71.4% at 773 K. Furthermore, conventional ZSM-5 showed a continuous decrease of its activity during subsequent TITB injection cycles, in which ca. 30% of the initial activity was lost after 30 instances of injection. On the other hand, no deactivation appeared in the case of meso ZSM-5 in the given 30 injections. Hence, meso-ZSM-5 shows enhanced anti-deactivation property over the conventional ZSM-5 catalyst. 

Among various BCP self-assembled structures, gyroid phases are very interesting (co)continuous 3D structures with great potential in various fields of applications, such as nanoporous membranes, solar cells, supercapacitors, and photonic crystals [35,36,37,38]. The single gyroid consists of the matrix phase and the continuous network (minor) phase, whereas the double gyroid has two inverted co-continuous network phases [39]. Taking the advantage of continuous nature of the matrix and network phases, fully interconnected nanochannels or a free-standing interconnected network can be obtained on the selective degeneration of the minor or matrix phase, respectively. For fabricating the mesoporous, inorganic materials with a precisely controlled texture, a sol-gel reaction [40], electroless plating [41], and electrochemical deposition [36,42,43] were performed within the BCP templates. 

**Figure 14:**
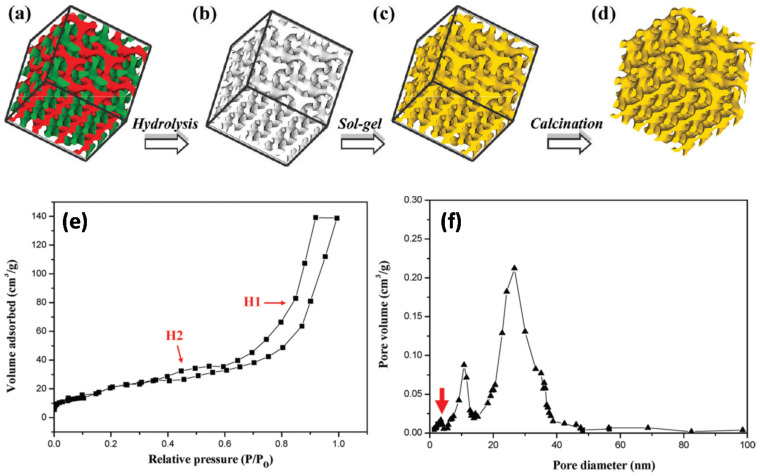
(**a**–**d**) Schematic illustration for the creation of well-defined, bicontinuous TiO_2_ from the templating of BCP: (**a**) PS–PLLA gyroid morphology (skeleton of double gyroid structure with two identical networks (green and red)); (**b**) gyroid-forming, mesoporous PS template after removal of minority PLLA networks. (**c**) PS/TiO_2_ gyroid nanohybrids via templated sol-gel reaction; (**d**) bicontinuous TiO_2_ with crystalline phase after removal of PS template through calcination. (**e**) N_2_ adsorption−desorption isotherm with hysteresis loops, indicating the mesoporous property of the bicontinuous TiO_2_. (**f**) Pore size distribution of the bicontinuous TiO_2_, calculated from the adsorption branch using the BJH method. Two major pore distributions with maxima around 11 and 24 nm pore size can be identified. Adapted from [44].

Huesh et al. reported the fabrication of a mesoporous polymers with gyroid nanochannels, employing the self-assembly of a PS–*b*–PLLA BCP with degradable PLLA block [44]. After the hydrolytic degradation of PLLA network phase, the mesoporous PS template was obtained and used as a template for sol-gel reaction to obtain a well-defined polymer/ceramic nanohybrid material with inorganic gyroid nanostructure. The morphology of the nanostructured materials was controlled by tuning the hydrolysis and condensation in the sol-gel reaction by adjusting the parameters, e.g., pH, temperature, and solvent conditions. After removal of PS and crystallization of TiO_2_ upon calcination, a bicontinuous TiO_2_ anatase phase was fabricated, which showed high photocatalytic activity for the decomposition of MB. This template approach contributed in the preparation of a self-supporting, crystalline, large-surface-area, and high-porosity TiO_2_ photocatalyst, which showed high photocatalytic activity for the decomposition of MB. Figure 14a–d shows the steps to fabricate crystalline, bicontinuous TiO_2_. Figure 14e shows the N_2_ adsorption desorption isotherm of bicontinuous TiO_2_, having two hysteresis loops (H1 and H2) in relative pressure regions from 0.35 to 1.00 Parallel adsorption–desorption lines in the H1 hysteresis loop indicate that bicontinuous TiO_2_ consists of ordered texture with uniform pores. Bicontinuous TiO_2_ comprised micropores sized below 5 nm, as shown by a red arrow in Figure 14f. The measured, specific surface area and porosity of bicontinuous TiO_2_ from BET are 257 m^2^g^−1^ and 51%, respectively, which were substantially higher than regular mesoporous TiO_2_ and responsible for the efficient photocatalytic activity. 

**Figure 15:**
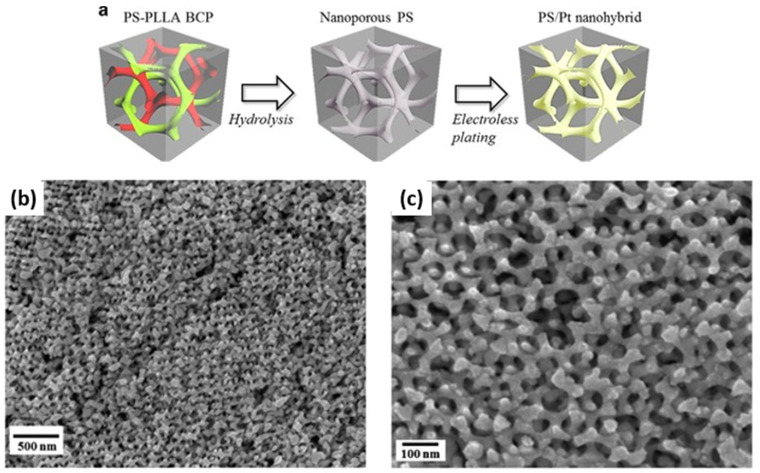
(**a**) Schematic illustration for the fabrication of the PS/Pt gyroid nanohybrids, using a BCP template from the hydrolysis of PS–*b*–PLLA for electroless plating. (**b**,**c**) FESEM micrographs of the nanoporous gyroid Pt from the PS/Pt gyroid nanohybrids after removal of the PS templates by UV degradation. Adapted from [45].

Cheng et al. reported the fabrication of nanoporous Pt with a gyroid nanostructure using a PS–*b*–PLLA block copolymer template, as schematically shown in Figure 15a [45]. The nanoporous template was also fabricated employing the self-assembly of degradable PS–*b*–PLLA BCP, followed by the hydrolysis of PLLA blocks. Next, the electroless plating was conducted to create a highly crystalline Pt gyroid phase in the PS matrix. The PS matrix was subsequently removed using UV exposure. Consequently, a nanoporous and interconnected gyroid Pt nanostructure was obtained and further used for electrochemical catalysis. Figure 15b,c shows the FESEM images of a nanoporous Pt with unique gyroid textures as a highly defined network with precisely controlled pore geometry.

**Figure 16:**
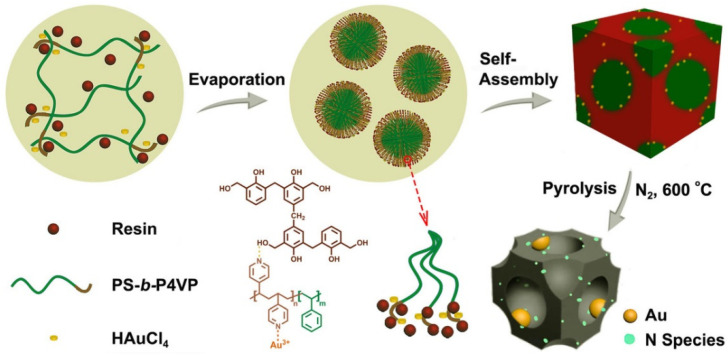
Synthesis approach of N-modified Au NPs encapsulated in ordered mesoporous carbon, with large pores employing self-assembly of a PS–*b*–P4VP diblock copolymer template. Adapted from [46].

Li et al. developed another approach that combines a self-assembled PS–*b*–P4VP template coordinated with Au ions and a phenolic resin, in order to synthesize N-modified Au nanoparticles in an ordered, mesoporous carbon matrix [46]. The scheme of this approach is shown in Figure 16. These nanostructures were synthesized by taking the advantage of the following factors: (1) the bond between the pyridine group with Au precursor; (2) the swelling of the P4VP segment by phenolic resin species, due to the hydrogen bonding; and, (3) the formation of monodisperse, PS–*b*–P4VP globular micelles via self-assembly. To obtain Au-decorated, black mesoporous carbon, an as-made sample was pyrolyzed in N_2_. During pyrolysis, the Au precursor was reduced to about 4 nm-sized, metallic Au NPs embedded in ordered mesoporous carbon matrix. By varying the length of the PS segment, the pore size could be tuned from 6 to 12 nm. Authors use this material as a catalyst to covert benzyl alcohol to benzoic acid, which showed good results. No loss of catalytic activity and leaching of Au nano catalyst was observed after 10 cycles.

**Figure 17:**
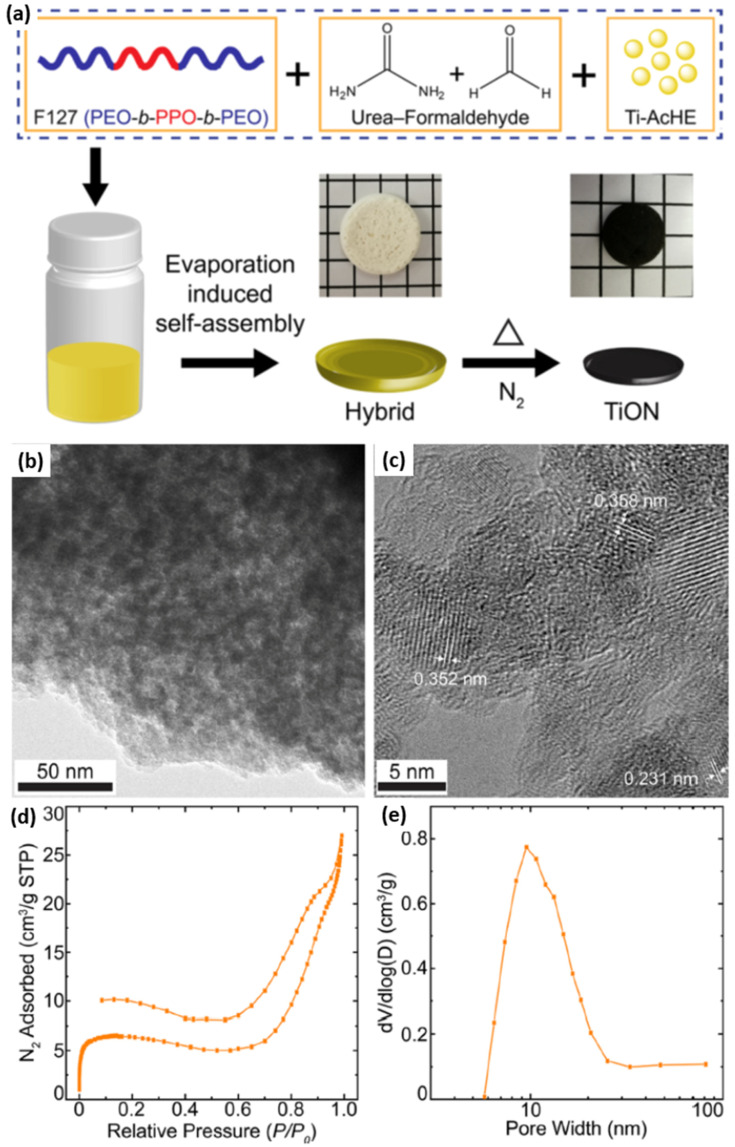
(**a**) Schematic of block copolymer-directed, mesoporous titanium oxynitride structures. Optical images (middle row) show the freestanding monoliths of the F127/urea−FA/Ti-AcHE hybrid composite (white-colored) and thermally annealed mesoporous TiON (black-colored), against 5 mm grid markings. (**b**) Bright-field TEM and (**c**) high-resolution TEM images of mesoporous crystalline TiON with the wormhole-like structure. (**d**) N_2_ sorption isotherm and (**e**) BJH pore size distribution of mesoporous crystalline TiON. Adapted from [47].

Using structure-directing BCPs, ordered mesoporous metal nitrides of targeted morphology and dimensionality could also be obtained. Recently, Li et al. presented an inexpensive method to fabricate highly ordered, crystalline, mesoporous, metal oxynitride structures that show an excellent electrochemical catalytic activity for hydrogen evolution process in KOH aqueous media [47]. The reported approach relied on a one-pot, ammonolysis-free soft templating synthesis, which was the first of its kind. In particular, the authors combined BCP-assisted self-assembly with metal sol and urea precursors to synthesize such nanostructures. A structure-directing ABA triblock copolymer (Pluronic F127), titanium-oxo-acetate sol nanoparticles (metal source), and oligomeric urea-formaldehyde (nitrogen source) were mixed to prepare hybrid monoliths by solvent evaporation-induced self-assembly. Hybrid monoliths were further pyrolyzed in a nitrogen environment to obtain crystalline, mesoporous titanium oxynitride (TiON) monoliths. A urea-formaldehyde additive was essential to provide nitrogen and support to the mesostructure upon crystallization during pyrolysis. The whole synthetic procedure is schematically shown in Figure 17a. TEM studies validated the withholding of the wormhole-like mesostructure after pyrolysis (Figure 17b). The presence of carbonaceous residue is responsible for the grainy texture of the TEM image. The lattice fringes of different crystallite orientations in the HR-TEM image (Figure 17c) confirmed the conversion of hybrids into highly crystalline, mesoporous structures. The porosity studies of the mesoporous, crystalline TiON structures revealed two sharp adsorption steps, attributed to a capillary condensation of nitrogen in the micropores and mesopores, respectively (Figure 17d,e). 

**Figure 18:**
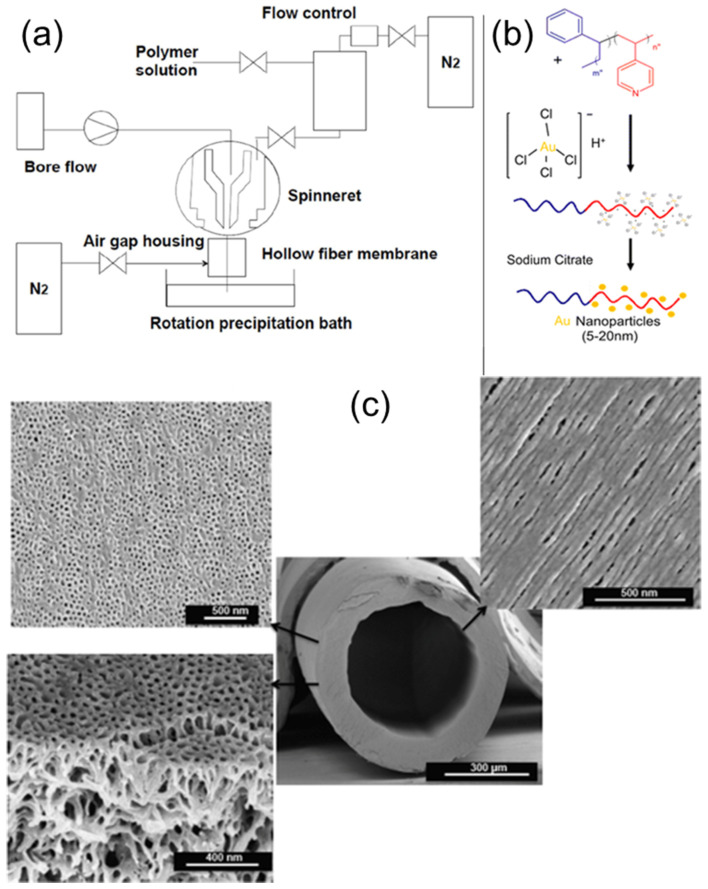
(**a**) Hollow fiber dry/wet spinning set-up. (**b**) Preparation of catalytic membranes by treatment with gold salts, followed by reduction with sodium citrate. (**c**) SEM image of PS-*b*-P4VP hollow fiber prepared via the dry/wet spinning method, with magnified SEM images of the outer and inner surface morphologies, as well as internal membrane morphology. Adapted from [48].

Combining the BCP self-assembly process with the dry/wet spinning technique, Hilke et al. prepared pH-responsive, PS–*b*–P4VP, hollow-fiber membranes with shell-side uniform pores, as schematically shown in Figure 18a [48]. The hollowness of the fibers was achieved by pushing a nonsolvent or water through the centre of the spinneret nozzle as a bore fluid. With the aid of a rotation precipitation bath, a porous, spongelike structure can be achieved from these fibers. A gold nano-catalyst was subsequently deposited onto the outer surface of hollow nanofibers, either by filtering presynthesized colloidal Au, or by reducing the Au ions after adsorption of the precursor. The single, Au-decorated hollow fiber was then tested against the catalytic reduction of 4-nitrophenol. 

## 3. Concluding Remarks and Outlook

The present review shows that significant progress has been made in preparing a broad range of nano-catalysts from self-assembled block copolymer templates. Block copolymer templates assist in fabricating “soft”, fragile matter, and allow for the generation of mesostructured, durable, and mechanically stable "hard" matters of a desired shape and order, further used for catalytic applications. The technique’s exclusivity lies in the robustness and ease with which precisely controlled catalytic nanostructures can be fabricated. The present review summarizes the approaches that have been used until now for fabricating such catalytic nanostructures, using a block copolymer self-assembly. BCP micellar template provided a cheaper and easier option to accommodate various precursors and presynthesized nano-catalysts in the desired domain of well-ordered BCP micelles decorated on the substrate. Furthermore, these catalysts were used for single and multiwalled CNT growth on the substrate and the oxidation of carbon monoxide by prepared catalysts. Similarly, inverted micellar arrays were fabricated by simple exposure to a suitable solvent. These inverted micellar arrays were used to fabricate bimetallic alloy catalysts composed of PtAu, PtIr, and AuAg. Another advancement was achieved in the shape of the catalyst. Au–CeO_2_ strawberry-like and Janus nanoparticles are excellent states of the art achieved by the BCP micellar template. The co-electrodeposition of Cu, Ni, and Au precursors into polymeric micelles resulted in a trimetallic mesoporous electrocatalyst, which showed high electrocatalytic activity and stability in the methanol oxidation reaction. Mesoporous Au–SiO_2_ yolk-shell nanoparticles were fabricated using PS–*b*–P4VP micelles as a sacrificial template, subjected to reduced 4-NP and Congo red dye. Yolk-shell nanostructures from BCP templates are interesting morphology in the field of catalysis to explore in the future. In the future, it will be interesting to see the effect of changing the shell component with some catalytically active material, such as TiO_2_, to achieve synergistic effects. Researchers synthesized ordered, mesoporous carbon materials doped with iron and nitrogen species by employing the crosslinked. self-assembled BCP template. By assisting post-steaming treatment, a mesoporous zeolite was prepared by direct synthetic method, using conventional BCP templates. Also, a BCP gyroid template-based, well-defined, bicontinuous TiO_2_ photocatalyst and nanoporous gyroid platinum nano-catalyst were prepared and further used for the decomposition of MB dye and electrochemical catalysis, respectively. Mesoporous titanium oxynitride structures were also fabricated using structure-directing ABA triblock copolymer (Pluronic F127), which shows excellent electrochemical catalytic activity for the hydrogen evolution process. The reported approach relies on a one-pot, ammonolysis-free, soft templating synthesis, which was the first of its kind. Researchers combined the BCP self-assembly process with the dry/wet spinning technique and prepared pH-responsive, PS–*b*–P4VP, hollow-fiber membranes with shell-side uniform pores used in the 4-nitrophenol reduction reaction.

As described in the current review, BCP templates could provide interesting methods to fabricate completely novel, mesoporous nano-catalysts, such as ternary alloy film, yolk-shell nanoparticles, iron-doped carbon, zeolite, bicontinuous TiO_2_ and gyroid platinum, and titanium oxynitride structures, which were further used as an efficient catalyst. It could be expected in the near future that significant technological improvements will be achieved from combining metal nano-objects and inorganic materials. In the future, a multifunctional hybrid nano-catalyst with different supports exhibiting synergistic effects can be prepared using these BCP templates. The scaling-up and economics of the process involved will need more attention in the future for its commercial viability. The focus area in terms of industry usage can be wastewater treatment (treatment of textile waste effluent via dye degradation), catalytic conversion of carbon monoxide, and methanol oxidation reactions.

## Data Availability

Not Applicable.
